# C-reactive Protein/Albumin Ratio as a Prognostic Indicator for Predicting Surgical Intervention in Neonates With Necrotizing Enterocolitis: A Prospective Cohort Study

**DOI:** 10.7759/cureus.87308

**Published:** 2025-07-04

**Authors:** Ahsan Ali Ghauri, Zubair Shaukat, Aziz Ahmad Chattha, Farrakh Mahmood Star, Armaghan Ahmed, Wajeeh U Rehman, Muhammad Ali Ghouri, Muhammad Saleem

**Affiliations:** 1 Pediatric Surgery, The Children's Hospital, University of Child Health Sciences, Lahore, PAK; 2 Physiology, Rahbar Medical and Dental College, Lahore, PAK

**Keywords:** albumin ratio, c reactive protein, necrotizing enterocolitis, neonatal inflammation, neonatal intensive care, neonatal surgery, prognostic biomarkers, surgical intervention

## Abstract

Background

Necrotizing enterocolitis (NEC) is a major cause of neonatal gastrointestinal morbidity and mortality. Early prediction of the need for surgical intervention remains challenging due to reliance on late clinical or radiological signs. The C-reactive protein and albumin ratio (CRP/Albumin), reflecting both inflammation and nutritional status, may serve as a dynamic biomarker to guide timely surgical decision-making.

Objective

The main objective of this study is to evaluate the prognostic utility of the CRP/Albumin ratio, and its temporal progression over the first three days of illness, in predicting surgical intervention and mortality in neonates.

Methods

In this prospective cohort study at a tertiary care hospital, Lahore, 66 neonates diagnosed with Bell’s Stage I-IIIa NEC were enrolled over a year. CRP and albumin levels were measured daily for three consecutive days, and the CRP/Albumin ratio was calculated. Patients were grouped into progression trends: decreasing, increasing, stable, or variable. Outcomes included surgical intervention and mortality. Statistical analysis included receiver operating characteristic (ROC) curve analysis, sensitivity, specificity, and comparative group analysis.

Results

Among 66 neonates (mean gestational age: 37.2 ± 3.2 weeks), 42.4% required surgery. Neonates with an increasing CRP/Albumin ratio trend had a significantly higher rate of surgical intervention (89.5%) and mortality (26.3%) compared to those with a decreasing trend (8.1% surgery and 2.6% mortality) (p < 0.001). The CRP/Albumin ratio demonstrated excellent predictive accuracy for surgery, with area under the curve (AUC) values of 0.926, 0.954, and 0.959 on Days 1-3, respectively. Optimal cut-offs on Days 2 and 3 yielded sensitivities and specificities above 85%. Serial monitoring of the ratio outperformed single-time-point measurements in prognostic value.

Conclusions

CRP/Albumin is a simple, reliable, and cost-effective biomarker that can lead to early identification of neonates at risk of surgical intervention and poor outcomes in NEC. Monitoring its temporal progression, rather than relying on single measurements, appears to enhance its predictive accuracy and may support timely decision-making in clinical settings.

## Introduction

Necrotizing enterocolitis (NEC) remains a leading cause of gastrointestinal morbidity and mortality among neonates, particularly in preterm and very low birth weight neonates [1-3]. Despite advances in neonatal intensive care, determining the optimal timing for surgical intervention remains challenging, often relying on conventional clinical and radiographic signs that may appear late in the disease course [4-6]. Early surgical decision-making is critical, as delayed intervention has been associated with bowel necrosis, perforation, spillage of intestinal contents, and peritonitis, leading to multiorgan failure - thus increasing mortality to as high as 19% ​[5-8]. 

The inflammatory cascade in NEC involves a systemic inflammatory response (SIRS). C-reactive protein (CRP), a well-known acute-phase protein, increases in response to ongoing inflammation but is non-specific [9]. Conversely, serum albumin reflects both nutritional status and capillary leak, often indicating systemic compromise in critically ill neonates [10]. The CRP/Albumin ratio has emerged as a promising biomarker that integrates inflammatory and nutritional aspects. Its prognostic value has been widely studied in the adult population in various clinical conditions, including sepsis, community-acquired pneumonia, and inflammatory bowel disease [11,12]. In neonates, preliminary evidence suggests that this ratio may have prognostic value in critically ill patients [13,14]. However, its application in NEC is still emerging and limited due to its dynamic changes over time in relation to clinical outcomes [15].

This study aims to evaluate the prognostic utility of the CRP/Albumin ratio, and particularly its temporal progression over the first three days of illness, in predicting surgical intervention and mortality in neonates diagnosed with NEC.

## Materials and methods

This prospective cohort study was conducted at the Department of Pediatric Surgery, The Children's Hospital, University of Child Health Sciences, Lahore, Pakistan. Neonates diagnosed with NEC and admitted between July 2023 and July 2024 were enrolled to evaluate the prognostic value of the CRP/Albumin ratio in predicting surgical intervention and mortality, after obtaining informed consent from parents or legal guardians. Ethical approval was obtained from the Institutional Review Board (IRB) of the University of Health Sciences, Lahore (approval no. UHS-Education/126-23). 

A total of 66 neonates were included in the study based on the inclusion criteria. The inclusion criteria consisted of neonates aged 0-28 days, diagnosed with NEC at Bell’s Stage I, II, or IIIa. Exclusion criteria included neonates with Bell’s Stage IIIb (pneumoperitoneum), very low birth weight (<1500 g), congenital anomalies, severe comorbidities, metabolic disorders, or incomplete clinical or laboratory data. 

For each neonate, CRP and albumin levels were measured on three consecutive days: Days 1-3 of admission. The CRP/Albumin ratio was calculated daily using the formula: CRP (mg/L) divided by albumin (g/dL). In addition to this, clinical data were recorded, including age, gestational age, birth weight, NEC stage, surgical or conservative management, duration of hospital stay, and mortality during hospital stay. Surgical intervention was defined as operative management, typically indicated in cases with radiological or clinical evidence of pneumoperitoneum. 

Neonates were grouped according to the progression pattern of their CRP/Albumin ratio over the three-day period to determine whether changes in this marker could predict outcomes more accurately than single measurements. The flexible, progression-based classification included four groups: Group A, a consistently decreasing ratio; Group B, a consistently increasing ratio; Group C, a stable ratio with less than 10% variation; and Group D, a variable ratio without a clear trend. 

Data were analyzed by IBM SPSS Statistics for Windows, Version 29 (Released 2023; IBM Corp., Armonk, NY, USA). Descriptive statistics were presented as means ± standard deviation for continuous variables, and as frequencies and percentages for categorical variables. Comparative analysis of the CRP/Albumin ratio among the four progression groups was performed using one-way analysis of variance (ANOVA) or the Kruskal-Wallis test, depending on data distribution. Comparative analysis of the CRP/Albumin ratio, surgical intervention, and mortality was performed using the Chi-square or Fisher’s Exact test, as appropriate. Specificity, sensitivity, positive predictive value (PPV), and negative predictive value (NPV) were calculated for each progression pattern in predicting surgical intervention on Days 1-3. Receiver operating characteristic (ROC) curve analysis was used to assess the predictive performance of the CRP/Albumin ratio for surgical intervention, with the area under the curve (AUC) reported. 

## Results

The study included 66 neonates, with a mean gestational age of 37.18 ± 3.18 weeks (range: 30-41 weeks). The mean age at presentation was 3.15 ± 0.81 days. Hospital stay duration ranged from 4 to 13 days, with a mean of 7.59 ± 2.44 days. The mean CRP/Albumin ratio was 9.59 ± 9.60 on Day 1 (range: 1.50-26.00), 9.96 ± 10.67 on Day 2 (range: 1.19-27.00), and 10.33 ± 11.62 on Day 3 (range: 1.03-29.00) (Table 1).

**Table 1:** Baseline Characteristics of Neonates With Necrotizing Enterocolitis by CRP/Albumin Ratio CRP: C-reactive protein

Descriptive Statistics					
	N	Minimum	Maximum	Mean	SD
Gestational Age (Weeks)	66	30	41	37.18	3.181
Age (Days)	66	2	4	3.15	0.808
Hospital Stay (Days)	66	4	13	7.59	2.443
CRP/Albumin Ratio (Day 1)	66	1.50	26.00	9.5917	9.59667
CRP/Albumin Ratio (Day 2)	66	1.19	27.00	9.9638	10.67327
CRP/Albumin Ratio (Day 3)	66	1.03	29.00	10.3270	11.62266
Valid N (Listwise)	66	-	-	-	-

**Figure 1:**
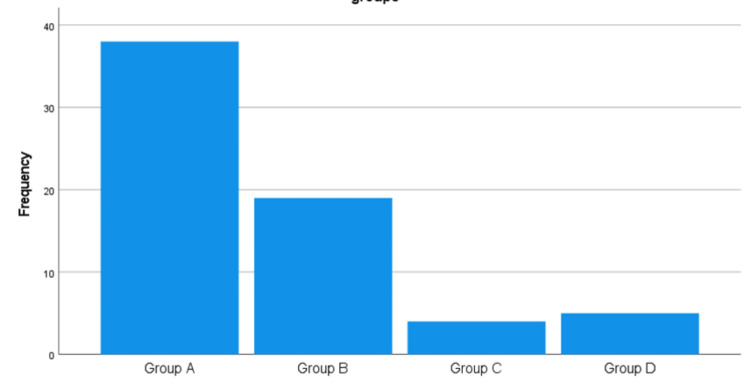
Distribution of Cases Among CRP/Albumin Progression Groups Bar chart showing the number of neonates in each CRP/Albumin progression group: Group A (decreasing trend, n = 37), Group B (increasing trend, n = 19), Group C (stable trend, n = 4), Group D (variable trend, n = 6). CRP: C-reactive protein

Patients were stratified into four groups based on the trend of CRP/Albumin ratio progression over three days: Group A (decreasing trend, n = 37), Group B (increasing trend, n = 19), Group C (stable trend, n = 4), and Group D (variable trend, n = 6) (Figure 1).

Comparative analysis of the CRP/Albumin ratio among progression groups on Days 1-3 showed a statistically significant difference across all three days. The Kruskal-Wallis H value for Day 1 was 41.260 (df = 3, p < 0.001), for Day 2, 38.295 (df = 3, p < 0.001), and for Day 3, 37.241 (df = 3, p < 0.001), indicating a significant progression pattern among the groups (Table 2).

**Table 2:** Comparison of CRP/Albumin Ratio Among Progression Groups on Days 1-3 Kruskal-Wallis test applied due to non-normal distribution of data, as assessed by the Shapiro-Wilk test. CRP: C-reactive protein

	Groups	N	Kruskal-Wallis H	df	Asymptotic Significance
CRP/Albumin Ratio (Day 1)	Group A	37	41.26	3	0.001
	Group B	19			
	Group C	4			
	Group D	6			
	Total	66			
CRP/Albumin Ratio (Day 2)	Group A	37	38.295	3	0.001
	Group B	19			
	Group C	4			
	Group D	6			
	Total	66			
CRP/Albumin Ratio (Day 3)	Group A	37	37.241	3	0.001
	Group B	19			
	Group C	4			
	Group D	6			
	Total	66			

Among 66 neonates, 38 (57.6%) responded successfully to conservative medical management, while 28 (42.4%) ultimately required surgical intervention. In Group A, 34 (89.5%) were successfully managed conservatively, and only three (10.5%) required surgery. In Group C, all four patients (100%) responded to conservative management. In contrast, among those in Group B and Group D, no patient responded to conservative medical management, and all underwent surgery. The association between CRP/Albumin progression and response to medical management was statistically significant (p < 0.001) (Table 3).

**Table 3:** Results of CRP/Albumin Ratio Progression and Conservative Management CRP/Albumin ratio trends over three days are shown in relation to the success of conservative management in NEC cases. CRP: C-reactive protein; NEC: necrotizing enterocolitis

			Conservative Medical Management		Total	Pearson Chi-Square	p-value
			Successful	Unsuccessful			
CRP/Albumin Ratio Progression	Decreasing	Count	34	3	37	54.714	0.000
		Expected Count	21.3	15.7	37.0		
	Increasing	Count	2	17	19		
		Expected Count	10.9	8.1	19.0		
	Stable	Count	4	0	4		
		Expected Count	2.3	1.7	4.0		
	Variable	Count	0	6	6		
		Expected Count	3.5	2.5	6.0		
Total		Count	38	28	66		
		Expected Count	38.0	28.0	66.0		

Among the 28 patients who required surgical intervention, the majority belonged to Group B (n = 17, 60.7%), followed by Group D (n = 6, 21.4%) and Group A (n = 3, 10.7%). None of the patients in Group C (n = 4, 10.5%) underwent surgery. Of the 38 patients who did not require surgery, most were from Group A (n = 34, 89.5%), with the remainder in Group C (n = 4, 10.5%). The association between CRP/Albumin progression and the need for surgical intervention was also statistically significant (p < 0.001) (Table 4).

**Table 4:** Results of CRP/Albumin Ratio Progression and Surgical Intervention This table shows the relationship between CRP/Albumin ratio trends and surgical intervention rates in NEC patients. CRP: C-reactive protein; NEC: necrotizing enterocolitis

			Surgical Intervention		Total	Pearson Chi-Square	p-value
CRP/Albumin Ratio Progression	Decreasing	Count	34	3	37	46.958	Asymptotic Significance (Two-Sided)
		Expected Count	22.4	14.6	37.0		0.001
	Increasing	Count	2	17	19		
		Expected Count	11.5	7.5	19.0		
	Stable	Count	4	0	4		
		Expected Count	2.4	1.6	4.0		
	Variable	Count	0	6	6		
		Expected Count	3.6	2.4	6.0		
Total		Count	40	26	66		
		Expected Count	40.0	26.0	66.0		

The mean hospital stays also varied significantly between groups (p < 0.001). Group A and Group C had the shortest hospital stays (7.00 ± 1.12 days and 7.00 ± 0.00 days, respectively), whereas Group B and Group D had longer stays (8.89 ± 2.81 and 11.60 ± 3.13 days, respectively).

Of the 66 neonates, eight patients (12.1%) died during treatment, while 59 (89.4%) were discharged. Mortality varied significantly across CRP/Albumin progression groups. In Group A, one patient (2.6%) died, while 37 (97.4%) survived. In contrast, in the increasing ratio Group B, five patients (26.3%) died. There was no mortality in Group C, while two patients (25%) died in Group D. Although the overall association between CRP/Albumin progression and mortality was not significant (p = 0.067), subgroup analysis showed significant mortality in Group A (p = 0.003) and Group B (p = 0.012). No statistical difference was noted between Group C and Group D due to the small number of cases in each group (Table 5).

**Table 5:** Clinical Outcomes by CRP/Albumin Ratio Progression Groups Outcomes, including discharge rate, mortality, and hospital stay, are compared across CRP/Albumin ratio progression groups. Significant differences are noted where applicable. CRP: C-reactive protein

		Discharge	Outcome Mortality					Total		Hospital Stay in Days		
		N	%	Sig.	N	%	Sig.	N	%	Mean	Std. Deviation	Sig.
Groups	Group A	37	62.70%	0.003	1	14.30%	0.003	38	57.60%	7	1.12	<0.001
	Group B	14	23.70%	0.015	5	71.40%	0.012	19	28.80%	8.89	2.807	
	Group C	4	6.80%	-	0	0.00%	-	4	6.10%	7.01	0	
	Group D	4	6.80%	-	2	14.30%	-	5	7.60%	11.6	3.13	
Total		59	100.00%		8	100.00%		66	100.00%	7.59	2.443	

The diagnostic performance of CRP/Albumin in predicting surgical intervention in neonates with NEC was assessed on Days 1-3. The AUC was 0.926 (95% CI: 0.860-0.992) for Day 1, 0.954 (95% CI: 0.903-1.005) for Day 2, and 0.959 (95% CI: 0.915-1.003) for Day 3 (p < 0.001 for all), indicating progressively higher predictive accuracy over time (Table 6).

**Table 6:** Area Under the Curve Values for CRP/Albumin Ratio Predicting Outcomes This table displays AUC values for the CRP/Albumin ratio over Days 1-3, in predicting surgical intervention in NEC patients. CRP: C-reactive protein; AUC: area under the curve; ROC: receiver operating characteristic; NEC: necrotizing enterocolitis

Area Under the ROC Curve					
Test Result Variable(s)	Area	Standard Error	Asymptotic Significance	Asymptotic 95% Confidence Interval	
				Lower Bound	Upper Bound
CRP/Albumin Ratio (Day 1)	0.926	0.034	0.000	0.860	0.992
CRP/Albumin Ratio (Day 2)	0.954	0.026	0.000	0.903	1.005
CRP/Albumin Ratio (Day 3)	0.959	0.023	0.000	0.915	1.003

**Figure 2:**
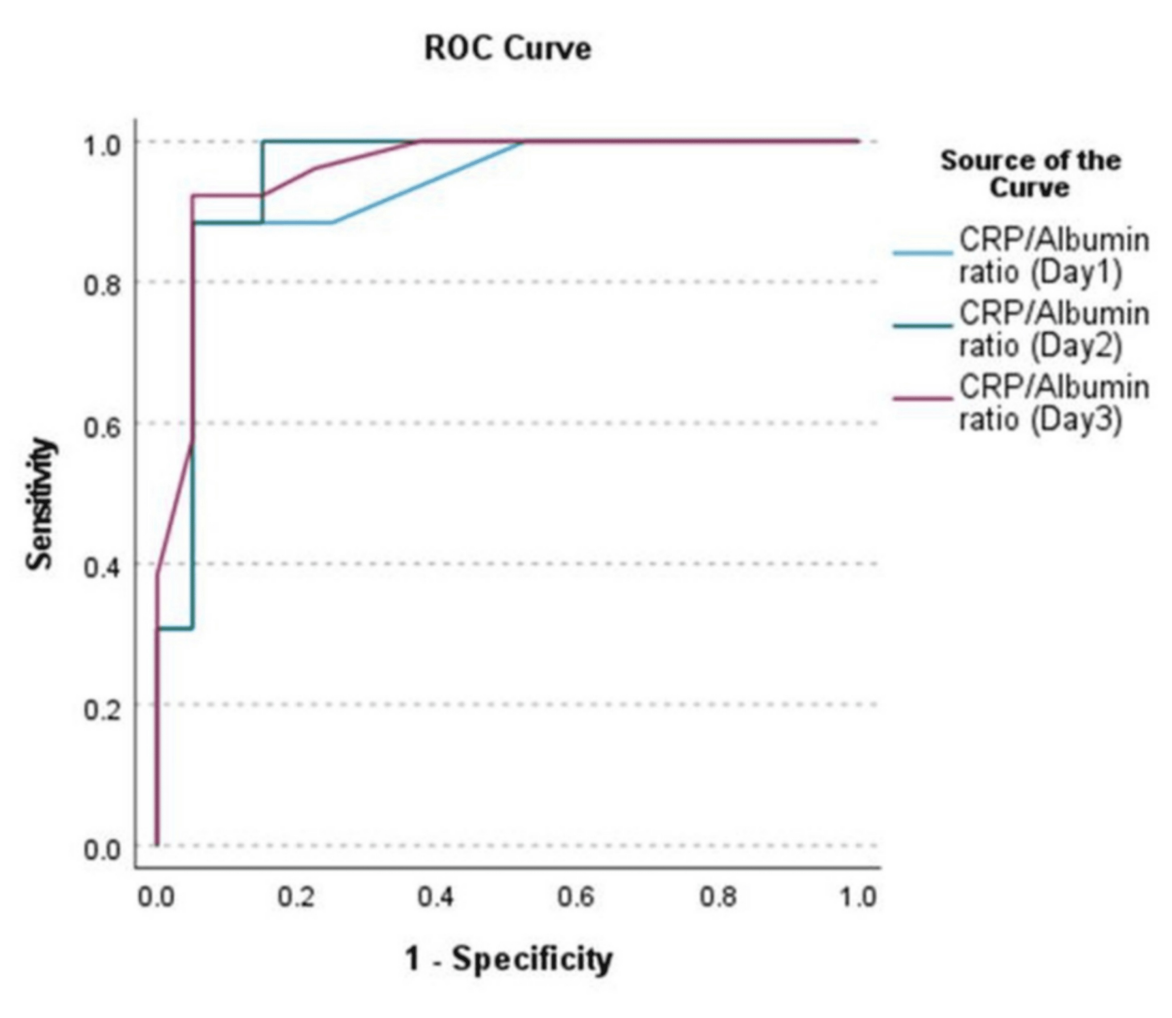
ROC Curve of CRP/Albumin Ratio on Days 1-3 for Predicting Surgical Intervention in NEC These results demonstrate the improving discriminative ability of the CRP/Albumin ratio over time. CRP: C-reactive protein; NEC: necrotizing enterocolitis; ROC: receiver operating characteristic

Optimal cut-off values were identified on the ROC curve and precision-recall curve. On Day 1, a CRP/Albumin ratio cut-off of 10.25 yielded a sensitivity of 88.5% and a specificity of 95.0%. For Day 2, a cut-off value of 2.05 achieved a sensitivity of 96.2% and a specificity of 85.0%. On Day 3, a cut-off of 2.31 provided both high sensitivity (92.3%) and high specificity (95.0%). The precision-recall analysis demonstrated high precision values (>0.90) at the corresponding optimal cut-offs, with Day 3 showing both a precision and recall of 92.3% (Figure 2).

**Figure 3:**
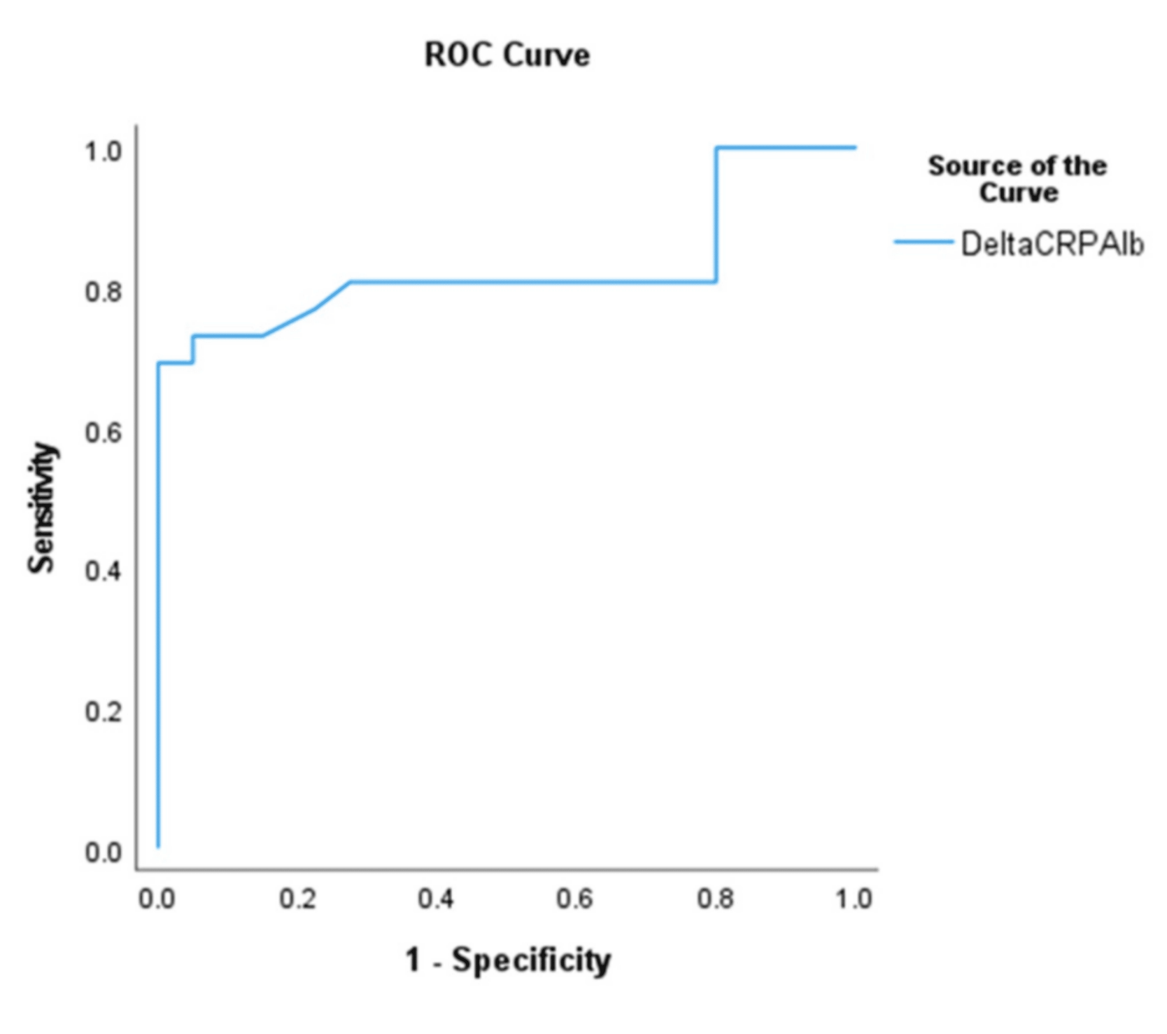
ROC Curve for Delta CRP/Albumin Ratio (Day 3 - Day 1) in Predicting Surgical Intervention in NEC Changes in CRP/Albumin ratio over three days (delta CRP/Albumin ratio) were categorized as increasing, decreasing, stable (<10% change), or variable. This ROC curve illustrates the predictive performance of the delta CRP/Albumin ratio over three days for surgical intervention in NEC patients. An increasing delta (change) indicates worsening inflammation and higher surgical risk, while a decreasing or stable delta suggests clinical improvements. CRP: C-reactive protein; NEC: necrotizing enterocolitis; ROC: receiver operating characteristic

The delta CRP/Albumin ratio also demonstrated an AUC of 0.827 at an optimal cut-off of -0.485, yielding a sensitivity of 80.8%, specificity of 62.5%, and a false positive rate of 27.5%. Notably, sensitivity remained 100% at a lower cut-off of -1.8, though with reduced specificity (Figure 3).

## Discussion

Early identification of neonates diagnosed with NEC who require surgery is crucial for improving outcomes [7,8,16,17]. This study assessed the prognostic value of the CRP/Albumin ratio and its dynamic/temporal progression over the first three days of illness to predict surgical intervention and mortality in NEC.

The overall incidence of surgical intervention and mortality is consistent with previous publications, reflecting the high burden of disease related to surgical intervention [2,15,18]. Importantly, our findings revealed that neonates with an increasing trend in CRP/Albumin ratio had higher rates of surgical intervention, prolonged hospital stay, and mortality, compared to those with a decreasing trend. This finding demonstrates the value of dynamic rather than static measurements of this marker, as serial monitoring captures the evolving inflammatory and nutritional status, reflecting disease progression more accurately. 

Previous studies have emphasized systemic inflammation and nutritional status as pivotal factors influencing NEC severity and prognosis [19-23]. While CRP and albumin have been studied individually, the combined CRP/Albumin ratio, monitored serially, appears to offer superior predictive accuracy [15]. To our knowledge, this is the first study from a resource-limited setting to evaluate the prognostic significance of CRP/Albumin ratio progression in NEC.

ROC curve analysis showed excellent predictive accuracy, with AUC values exceeding 0.9 from Day 1 to Day 3. The identified cut-off values yielded the best sensitivity and specificity, demonstrating that they can reliably guide clinical decision-making. Notably, the Day 2 and Day 3 cut-offs were lower than those on Day 1, reflecting the dynamic inflammatory course of NEC and potentially capturing earlier signs of disease progression.

The CRP/Albumin ratio was assessed only during the first three days of illness, focusing on early predictors of surgical need. Beyond this window, the biomarker’s trajectory may be influenced by interventions such as antibiotics, fluid resuscitation, nutritional support, or surgical intervention itself, thereby potentially confounding its prognostic value [9].

Although the overall association between CRP/Albumin ratio and mortality did not reach statistical significance, subgroup analyses revealed meaningful differences, with higher mortality observed in the increasing trend group than in the other subgroups. The absence of significant findings in the stable and varied trend groups may be attributed to limited sample size and low mortality, thereby reducing statistical power. Nonetheless, our data support previous studies showing that persistent systemic inflammation and poor nutritional status play a central role in NEC-related clinical deterioration [24].

This study has important clinical implications. Unlike many other advanced biomarkers, CRP and albumin testing are widely available, cost-effective, and can be routinely performed for early risk stratification in NEC, facilitating timely recognition of disease progression, earlier surgical intervention when needed, and, ultimately, improved neonatal outcomes.

Limitations 

This study has several limitations. Being a single center with a modest sample size, the generalizability of results is limited. Neonates with Bell's Stage IIIb and extremely low birth weight neonates, who are at high risk, were excluded, potentially introducing selection bias and affecting outcome estimates. Variability in clinical decision-making regarding surgical indications may have influenced the associations between biomarker trends and interventions. Additionally, although promising, the CRP/Albumin ratio was not compared directly with other established parameters such as white cell count, platelet count, electrolyte imbalances, or radiographic signs like pneumoperitoneum. These factors are important components of NEC assessment and could provide additional context for interpreting biomarker trends. Lastly, nutritional and inflammatory status can be influenced by factors unrelated to NEC, such as concomitant infections, prematurity, and intrauterine growth retardation - potential confounders that were not fully controlled for.

## Conclusions

In this prospective cohort study, serial CRP/Albumin ratio trends - particularly increasing values over 72 hours - strongly predict surgical intervention in NEC. This simple, reliable, and cost-effective biomarker can lead to early identification of neonates at risk of surgical intervention and poor outcomes in NEC. Monitoring its temporal progression, rather than relying on single measurements or specific cut-offs, enhances its predictive accuracy and may support timely decision-making in clinical settings. Furthermore, the delta CRP/Albumin ratio (change over time) demonstrates additional prognostic value, reinforcing the utility of trend analysis.

To confirm these findings and facilitate broader implementation, future multicenter studies with standardized protocols are needed to determine whether trend-based CRP/Albumin ratio monitoring can be formally incorporated into evidence-based NEC management guidelines.
